# Humanizing Stakeholders by Rethinking Business

**DOI:** 10.3389/fpsyg.2021.687067

**Published:** 2021-09-22

**Authors:** Katinka J. P. Quintelier, Joeri van Hugten, Bidhan L. Parmar, Inge M. Brokerhof

**Affiliations:** ^1^Department of Management & Organization, School of Business and Economics, Vrije Universiteit Amsterdam, Amsterdam, Netherlands; ^2^The Darden School of Business, The University of Virginia, Charlottesville, VA, United States; ^3^Faculty of Management Studies, The Open University of The Netherlands, Heerlen, Netherlands

**Keywords:** humanization, mind attributions, moral consideration, moral legitimacy, stakeholders, stakeholder orientation, profit orientation, business orientation

## Abstract

Can business humanize its stakeholders? And if so, how does this relate to moral consideration for stakeholders? In this paper we compare two business orientations that are relevant for current business theory and practice: a stakeholder orientation and a profit orientation. We empirically investigate the causal relationships between business orientation, humanization, and moral consideration. We report the results of six experiments, making use of different operationalizations of a stakeholder and profit orientation, different stakeholders (employees, suppliers, labor unions), and different participant samples. Our findings support the prediction that individual stakeholders observing a stakeholder-oriented firm see the firm’s other stakeholders as more human than individual stakeholders observing a profit-oriented firm. This humanization, in turn, increases individual stakeholders’ moral consideration for the firm’s other stakeholders. Our findings underscore the importance of humanization for stakeholders’ moral consideration for each other. This paper contributes to a deeper understanding of the firm as a moral community of stakeholders. Specifically, we move away from a focus on managers, and how they can make business more moral. Instead we direct attention to (other) stakeholders, and how business can make these stakeholders more moral.

## Introduction

In the 1936 movie Modern Times, Charlie Chaplin (1936) criticized modern business activity as dehumanizing (Vance, 2003). For instance, in the movie, the president of a factory plugs Charlie, the protagonist, into an automatic feeding chair so that Charlie can continue screwing bolts during lunch. Such scenes were inspired by a broader social concern that saw modern business management as antithetical to the broader interests of human beings (Flom, 1997, p. 80). Current scholars echo this criticism (Moore, 2005; Akrivou and Bradbury-Huang, 2015). For instance, stakeholder scholars argue that managers may fail to see and treat stakeholders as real people who deserve moral consideration (Donaldson, 1999; McVea and Freeman, 2005; Newkirk and Freeman, 2008; Freeman et al., 2010; Parmar et al., 2010; Purnell and Freeman, 2012). However, stakeholders who are not managers may also fail to see and treat each other as real people who deserve moral consideration, and such a situation can also have negative cognitive and emotional consequences for all involved (Bastian and Haslam, 2011). In this paper, we therefore redirect the focus from managers, to stakeholders who are not cast in a role as manager.

Does business lead to the dehumanization of stakeholders? Stakeholder scholars note that firms vary in their business orientation: Firms can be profit-oriented (maximizing short-term profit) or more stakeholder-oriented (balancing the interests of stakeholders). Stakeholder scholars also see links between a firm’s business orientation, humanization of stakeholders, and moral consideration for stakeholders (Freeman et al., 2010; Parmar et al., 2010). For instance, they argue that managers in profit-oriented firms may fail to see and treat stakeholders as real people who deserve moral consideration. In contrast, managers in stakeholder-oriented firms may see and treat stakeholders as human beings who deserve moral consideration (Freeman, 1994; McVea and Freeman, 2005; Harris and Freeman, 2008; Newkirk and Freeman, 2008; Freeman et al., 2010; Purnell and Freeman, 2012). However, it remains as yet unclear if the relationship would also work in other ways, specifically, if stakeholder-oriented firms would cause individual stakeholders, when they do not have a managerial role, to humanize and show moral consideration for other stakeholders.

To investigate the effect of a business orientation on humanization and moral consideration, we turn to the psychology of (de)humanization. (De)humanization is here defined as the attribution of more (or less) human-like qualities to human or non-human entities (Waytz et al., 2010a). Previous work finds that individual stakeholders can dehumanize other stakeholders (Christoff, 2014). Individuals outside a business context can also humanize members of another group than their own (Delgado et al., 2012; Davies et al., 2018). However, in a business context, empirical work on the causes of humanization of stakeholders, as opposed to the dehumanization of stakeholders, is to our knowledge missing. In addition, while there is a relation between (de)humanization and moral cognition (Gray et al., 2012), the exact causal relationship between humanization and moral cognition depends on the specific dimension of humanization and of moral cognition (Gray and Wegner, 2009). This necessitates a more thorough investigation of the causal relationships between a stakeholder orientation, humanization, and moral consideration in a business context.

To investigate these causal relationships, we conceptualize a stakeholder and a profit orientation in line with current theoretical (Freeman et al., 2020) and practical developments (Business Roundtable, 2019; Harrison et al., 2020). Based on the psychology of humanization (Epley et al., 2007; Waytz et al., 2010a), we first develop the hypothesis that individual stakeholders observing a stakeholder-oriented firm will humanize other stakeholders compared to individual stakeholders observing a profit-oriented firm. Second, we develop the hypothesis that humanization of stakeholders increases moral consideration for stakeholders. Third, on the basis of our first two hypotheses, we hypothesize that individual stakeholders observing a stakeholder-oriented firm will show more moral consideration for other stakeholders than individual stakeholders observing a profit-oriented firm, mediated by individual stakeholders’ humanization of other stakeholders. We find support for the first two hypotheses in six experiments, across different operationalizations of business orientations, different stakeholders, and different participant samples. We also find that the positive relationship between a stakeholder orientation and moral consideration only materializes if humanization is asked before moral consideration. It is then fully mediated by humanization. This supports the importance of humanization and its temporal precedence in increasing individual stakeholders’ moral consideration for each other.

This paper contributes to our understanding of the firm as a moral community of stakeholders. First, our work goes beyond a focus on managers as the locus of moral responsibility toward stakeholders. Specifically, we investigate when stakeholders who are not cast in a role as manager will show moral consideration for each other, which is essential to uphold ethical firms (Goodstein and Wicks, 2007). Relatedly, while scholars argue that individual stakeholders can make business more morally considerate (Newkirk and Freeman, 2008; Purnell and Freeman, 2012; Akrivou and Bradbury-Huang, 2015), we investigate if business can also increase individual stakeholders’ moral consideration. Second, our findings underscore the importance of humanization for increasing individual stakeholders’ moral consideration for each other. This sharpens previous accounts where humanization was not taken into account (e.g., Harris and Freeman, 2008; Bridoux and Vishwanathan, 2020).

## Theory and Hypotheses

Business scholars from a variety of disciplines advocate for the humanization of, and moral consideration for, stakeholders (Ferraro et al., 2005; Ghoshal, 2005; Moore, 2005; Newkirk and Freeman, 2008; Akrivou and Bradbury-Huang, 2015). One of these research disciplines, stakeholder theory, recently gained prominence. In 2019, 181 CEO’s signed a statement that from now on they would lead their companies for the benefit of all stakeholders, instead of putting (short-term) profit for shareholders first (Business Roundtable, 2019; Harrison et al., 2020). The discourse of their statement resembles theorizing by stakeholder scholars, who also advocate rethinking business from a short-term profit orientation to a stakeholder orientation (Freeman et al., 2010, 2020). Because of these current developments, we conceptualize a business orientation in line with stakeholder theory.

A business orientation is a firm-level construct (Berman et al., 1999; Jones et al., 2007; Freeman et al., 2010; Phillips et al., 2011; Bridoux and Vishwanathan, 2020). It refers to a set of prescriptions about the extent to which a firm balances the interests of a broad range of stakeholders or aims to maximize short-term profit (cf. Jones et al., 2007; Phillips, 2011; Freeman et al., 2020). We agree that stakeholder interests and profit can go together in the long term (Jones et al., 2018). However, while going together in the long term, profit for shareholders versus value for stakeholders can still be a zero-sum choice in the short term (Harrison and Bosse, 2013). In that case, firms can trade off stakeholder value for short-term profit, scoring high on either stakeholder or profit elements, and low on, respectively, profit or stakeholder elements (Brickson, 2005). The first five experiments in this paper describe firms that make this trade-off. This means that participants either saw a stakeholder-oriented firm that scores high on stakeholder elements and low on profit elements, or a profit-oriented firm that scores high on profit elements and low on stakeholder elements. Alternatively, also in the short term, firms can aim to find synergy (Tantalo and Priem, 2016) or win-win solutions (Dembek et al., 2015), scoring high on one element without scoring low on the other. In order to capture this possibility as well, in the last experiment participants either saw a stakeholder-oriented firm that scores high on stakeholder elements while profit elements are not manipulated; or a profit-oriented firm that scores high on profit elements while stakeholder elements are not manipulated. For both descriptions, when a firm scores high on stakeholder elements it is called stakeholder-oriented and when a firm scores high on profit elements it is called profit-oriented.

A firm is stakeholder-oriented when the firm’s focus is on balancing the interests of a broad range of stakeholders. This can lead to fair, transparent stakeholder interactions such as adopting a living wage for employees, giving voice to stakeholders, developing long-term and cooperative relationships, as well as contracting based on trust (Harrison et al., 2010; Bridoux and Stoelhorst, 2014). A firm is profit-oriented when the focus is on short-term profit maximization. This can lead to interactions with non-shareholder stakeholders on the basis of bargaining power (Jones et al., 2007; Phillips et al., 2011; Wickert et al., 2017; Parmar et al., 2019) such as aggressive contracting, hard bargaining, minimizing labor costs, playing stakeholders off against each other, replacing stakeholders and resolving problems through legal procedures (Brickson, 2005, 2007; Jones et al., 2007; Bridoux and Stoelhorst, 2014).

Stakeholder scholars argue that there is a relationship between a stakeholder orientation and managers’ humanization of, and moral consideration for stakeholders. However, next to managers, firms also consist of other stakeholders, who directly and indirectly interact with each other (Rowley, 1997). In these interactions, stakeholders can show more or less moral consideration for each other (Goodstein and Wicks, 2007; Bridoux and Vishwanathan, 2020). This moral consideration from individual stakeholders who are not *per se* managers, toward other stakeholders, has important consequences. For instance, moral consideration can motivate employees to speak up about organizational wrongdoing, or it can motivate consumers to cooperate with a recycling scheme reducing electronic waste (Goodstein and Wicks, 2007). In this paper, we specifically investigate the effect of a stakeholder orientation on individual stakeholders who are not cast in a role as manager. Specifically, we investigate the effect of a stakeholder orientation on individual stakeholders’ humanization of, and moral consideration for, other stakeholders. We speak of “individual stakeholders” when we refer to the individual stakeholders who are not *per se* managers, and who are (to some extent) humanizing or showing moral consideration, and we will speak of “other stakeholders” when we refer to the stakeholders who are the object of humanization and moral consideration.

### The Effect of Business Orientation on Humanization

Humanization is multidimensional, meaning that individuals in general can attribute a variety of human-like qualities to human or non-human entities (Waytz et al., 2010a). Attributions that are relevant for moral consideration are mind attributions (Gray et al., 2012), which consist of attributions of agency (the capacity for planning and actions) and experience (the capacity for feeling and emotions) (Haslam, 2006; Gray et al., 2007, 2012; Waytz et al., 2010b; Sherman and Haidt, 2011; Singer, 2011). In this paper, individual stakeholders are said to humanize stakeholders if they attribute more mind to these stakeholders.

In light of the research on humanization, we expect that individual stakeholders observing a stakeholder orientation will humanize other stakeholders, compared to individual stakeholders observing a profit orientation. The argument is that a stakeholder orientation, compared to a profit orientation, is more suggestive of stakeholders having mind, and individual stakeholders easily humanize entities on the basis of suggestions. Stakeholder-oriented firms tend to engage in practices that imply that stakeholders have a mind, such as resolving problems through collaboration with stakeholders (Bridoux and Stoelhorst, 2014), and supporting and upholding stakeholder rights (Bryher, 2019). In addition, stakeholder-oriented firms tend to engage in practices that highlight the experiences of stakeholders, such as accommodating their needs and showing loyalty (Brickson, 2005). Muller et al. (2013) point out that more stakeholder-oriented firms use more vivid references to human beings with needs, and elicit appraisals of how these human beings are affected by their plight. In contrast, profit-oriented firms tend not to interact with their stakeholders in a way that is suggestive of mind. Instead, firms emphasizing profit goals describe their stakeholder relations as “an instrument to improve [their] financial performance” (Maignan and Ralston, 2002, p. 501).

When observing interactions that are suggestive of an entity having mind, individuals in general attribute more human-like qualities to that entity. Seminal studies show that even simple shapes can be seen as more human when they interact with each other as if they have agency and experience (Heider and Simmel, 1944). Also, human beings are attributed higher or lower levels of mind depending on how their agency and experience are described. For example, Gray and Wegner (2009) described a person as either more or less sensitive to pain, and found that their research participants attributed a higher capacity for experience to the person who was described as being more sensitive to pain. In contrast, individual stakeholders who see other stakeholders as instruments to maximize profit are likely to attribute less mind to other stakeholders: Boyer and Petersen (2018) argue that most people perceive profit motives as impersonal (Boyer and Petersen, 2018). Similarly, when people have just eaten animals, i.e., used them as instruments to satisfy their hunger, they attribute less mind to animals (Bastian et al., 2012b). These arguments lead to the following hypothesis:

H1: Individual stakeholders who observe a stakeholder-oriented firm will humanize the firm’s other stakeholders compared to individuals who observe a profit-oriented firm.

### The Effect of Humanization on Moral Consideration

One of the central claims in stakeholder theory is that the interests of stakeholders should be seen as morally legitimate, this is, as morally valid, or as interests that should be taken into account (Donaldson and Preston, 1995; Mitchell et al., 1997; Agle et al., 1999; Donaldson, 1999; Phillips, 2003). This concept of moral legitimacy in stakeholder theory is closely related to concepts in psychology. For instance, in psychology, an entity has moral standing to the extent that the entity itself is deemed deserving of respect and moral consideration (cf. Piazza et al., 2014). Other studies in psychology speak of moral patiency, which is the extent to which moral rights or wrongs can be done to the entity (Gray et al., 2007). In this paper, we speak of moral consideration as a general term.

Humanization is multidimensional, and research suggests that some dimensions of humanization can elicit harsher moral judgments, rather than stronger moral consideration (Gray and Wegner, 2009; Haran, 2013; Haran et al., 2016). However, a body of research supports that mind attributions, as a specific dimension of humanization, are positively related to moral consideration (Gray et al., 2007). For instance, mind attributions to an entity are positively related to willingness to save an entity from destruction (Gray et al., 2007). This relation is even evident in early childhood (Sommer et al., 2019), and exists for entities ranging from non-living things and robots (Kahn et al., 2006, 2012), to animals (Bastian et al., 2012a) and human beings (Bastian et al., 2011).

Experimental evidence supports a causal relationship between mind attributions and moral consideration. Exemplifying this, the experimentally induced objectification of women elicits lower mind attributions than a control condition, and this causes lower moral consideration for these women (Loughnan et al., 2010). Similarly, reading about animals’ commonalities with humans increases mind attributions to animals, and this in turn increases moral concern for animal welfare (Bastian et al., 2012a). Experimentally manipulating participants’ cognition about people’s mental states also reduces participants’ willingness to sacrifice these people’s lives in a moral dilemma (Majdandžić et al., 2012). We therefore hypothesize that humanization of stakeholders causes moral consideration for these stakeholders.

H2: When individual stakeholders humanize other stakeholders, they will show more moral consideration for those other stakeholders.

Based on hypotheses 1 and 2, we infer that a stakeholder orientation will positively relate to moral consideration for stakeholders. We therefore expect a mediated effect of business orientation on moral consideration. This leads to the following hypothesis:

H3: Individual stakeholders who observe a stakeholder-oriented firm will show more moral consideration for the firm’s other stakeholders compared to individual stakeholders who observe a profit-oriented firm. This is mediated by individual stakeholders’ humanization of the firm’s other stakeholders.

## Overview of Experiments

In order to test causal relationships, vignette experiments are suitable, because vignettes allow to test causal relationships by manipulating the independent variable, while adding realism to the background description (Aguinis and Bradley, 2014). We developed vignettes that were short, carefully constructed scenarios describing a firm, representing a systematic combination of the elements of interest (Atzmüller and Steiner, 2010, p. 128), in this case focusing on stakeholder-oriented or profit-oriented elements. In the experiment, participants were cast in the role of customer – an external stakeholder – and asked about their humanization of and moral consideration for the firm’s other stakeholders.

Six experiments tested the theory, and replicated the findings across two operationalizations of a stakeholder and profit orientation, with different stakeholders (stakeholders in general, employees, labor unions, and suppliers), and with different participant samples. First, in experiment 1, we investigated the effect of a stakeholder versus profit orientation on individual stakeholders’ humanization of stakeholders in general. In experiments 2–6, we investigated the effect of a stakeholder versus profit orientation on individual stakeholders’ moral consideration for other stakeholders, mediated by humanization of these other stakeholders. We tested the specificity of our findings by testing humanization of, and moral consideration for, employees (exp. 2), suppliers (exp. 3), and labor unions (exp. 4). Next, experiment 5 provides further support for humanization as a mediator, by highlighting the importance of its temporal precedence. In experiment 6, we found support for our model when manipulating the firm’s orientation without a trade-off and without explicit references to morality. We used different participant samples to investigate the generalizability of our findings.

## Experiment 1

### Methods

#### Sample and Design

We expected a large effect size (Rai and Diermeier, 2015). According to a (conservative) calculation with the help of the power analysis program G^∗^Power (Faul et al., 2007), this requires a sample size of 84. One hundred ninety eight participants completed the experiment (109 men; *M*_*age*_ = 39.64; 100 stakeholder-oriented). After removing participants who gave a wrong answer to an attention check, 180 participants remained (96 men, *M*_*age*_ = 39.89, 96 stakeholder-oriented). Participants were recruited on www.clickworker.com – a German website similar to Amazon’s Mechanical Turk (MTurk) – and compensated 0.6€. Participants were randomly assigned to a stakeholder- or profit-oriented vignette, thus using a one-way between-subjects design. The vignette was followed by two attention check questions, and a survey probing for participants’ humanization of the stakeholders. This was followed by a manipulation check, control variables, and demographics.

#### Procedure

Participants were introduced to the experiment. We asked them to confirm that they had read the information and that they agreed to proceed. Participants were then instructed to carefully read the description of a hypothetical “grocery retailer Alpha, selling products that you buy on a weekly basis.” This means that participants were cast in the role of customer, an external stakeholder of Alpha. Alpha was either described as stakeholder-oriented or profit-oriented, based on the description in the Theory and Hypotheses section. The vignettes were similar in length and wording, only varying in phrases related to their orientation. The *stakeholder-oriented* (profit-oriented) vignette read:

Alpha is committed to improving its *stakeholders’ welfare* (financial performance), because Alpha believes this is *the morally right thing to do* (necessary to be a successful business). This commitment to *stakeholder welfare and doing what is morally right* (financial performance and being a successful business) translates into practices that improve *stakeholder welfare* (financial performance), also if these practices result in lower *financial performance* (stakeholder welfare). Specifically, Alpha *invests in relationships with its suppliers, rather than switching to the supplier who asks the lowest price* (switches to the supplier who asks the lowest price rather than investing in relationships with its suppliers). In addition, Alpha constantly optimizes its operations to increase *customer satisfaction, also if this leads to lower profits* (profits, also if this leads to lower customer satisfaction). When new skills are needed, Alpha *trains its current employees, instead of replacing them with skilled applicants who ask the same wage* (replaces its employees with skilled applicants who ask the same wage, instead of training current employees). Finally, Alpha resolves conflicts with the local community *through collaboration rather than via legal procedures* (via legal procedures rather than through collaboration).

After reading the vignette, participants completed two attention check questions, whereby participants were instructed to select the statement that appeared in the description of Alpha in the vignette. For the first question, the participants could choose between: “Alpha is a grocery retailer selling products that you buy on a weekly basis” (correct statement); “Alpha has job openings consistent with your career goals,” “Alpha is a corporation that you might include in your investment portfolio.” For the second question, participants could choose between “Alpha is committed to improving its financial performance, because Alpha believes this is necessary to be a successful business” (correct for profit-oriented), “Alpha is committed to improving its stakeholders’ welfare, because Alpha believes this is necessary to be a successful business” and “Alpha is committed to improving its stakeholders’ welfare, because Alpha believes this is the morally right thing to do” (correct for stakeholder-oriented). Participants who failed to correctly select each statement were removed from the analyses.

In order to measure humanization, mind attribution items from Waytz et al. (2010a) were adapted to fit an organizational context. Participants were asked to rate five items on a seven-point Likert scale from “*not at all*” to “*very much*.” The items were: “To what extent do Alpha’s stakeholders have intentions?”; “To what extent do Alpha’s stakeholders have free will?”; “To what extent do Alpha’s stakeholders have a mind of their own?”; “To what extent do Alpha’s stakeholders experience emotions?” and “To what extent do Alpha’s stakeholders have consciousness?”. The scale’s reliability was high (Cronbach’s alpha = 0.901).

Participants completed a manipulation check to see if participants indeed saw one vignette as more stakeholder-oriented and the other as more profit-oriented. Specifically, we asked participants to indicate on a slider scale from 0 to 100 to what extent they thought the firm was long-term versus short-term oriented, and to what extent the firm prioritized stakeholders versus firm-level performance. These features were not literally in the vignette description, but they are a more abstract description of the purpose and practices that describe a stakeholder orientation, as described in the Theory and Hypotheses section above. A pilot study on MTurk (*n* = 46) had revealed that participants indeed perceive the stakeholder-oriented vignette as more long-term oriented (*M* = 80.17; *SD* = 19.09) and prioritizing stakeholders (*M* = 75.17; *SD* = 20.11) than the profit-oriented vignette (long-term: *M* = 41.56; *SD* = 25.15; stakeholders: *M* = 31.95; *SD* = 23.51) and these differences were significant [long-term: *F*(1,44) = 44.87; *p* < 0.001; stakeholders: *F*(1,44) = 34.39; *p* < 0.001].

Finally, participants filled out demographic variables (year of birth, gender, nationality, level of education, and occupational status). As control variables, we asked participants to indicate to what extent the vignettes were realistic and imaginable (“The description was realistic,” and “I had no difficulty imagining this situation”).

#### Results and Discussion

As can be seen in Table 1, A MANOVA revealed that the stakeholder-oriented firm was perceived as significantly more long-term oriented (*M* = 75.14; *SD* = 19.45) than the profit-oriented firm [*M* = 36.88; *SD* = 27.11; *F*(1, 178) = 146.40; *p* < 0.001] and as significantly more prioritizing stakeholders (*M* = 76.65; *SD* = 19.43) than the profit-oriented firm [*M* = 32.87; *SD* = 24.63; *F*(1, 178) = 120.33; *p* < 0.001]. There were no differences in demographics between the conditions. However, the profit-oriented firm (*M* = 5.45; *SD* = 1.08) was perceived as significantly more realistic than the stakeholder-oriented firm [*M* = 4.94; *SD* = 1.22; *F*(1,178) = 8.877; *p* = 0.003]. We proceeded with the analysis but included realism as a control variable.

**TABLE 1:** Means, standard deviations, and significance levels of manipulation checks and control variables in experiments 1–6.

**Exp.**	**Variable**	**Stakeholder**	**Profit**	
		
		**M (SD)**	**M (SD)**	**F (p)**
1	Long-term	75.14 (19.45)	36.88 (27.11)	*F*(1, 178) = 146.40 (< 0.001)
	Stakeholders	72.65 (19.43)	32.87 (24.63)	*F*(1, 178) = 120.33 (< 0.001)
	Control: realism	4.94 (1.22)	5.45 (1.08)	*F*(1, 178) = 8.87 (0.003)
2	Long-term	76.47 (13.64)	28.41 (24.55)	*F*(1, 181) = 263.88 (< 0.001)
	Stakeholders	74.39 (17.31)	23.81 (23.15)	*F*(1, 181) = 277.78 (< 0.001)
	Control: age	32.21 (14.41)	27.78 (10.94)	*F*(1, 181) = 5.51 (0.020)
3	Long-term	73.82 (16.65)	28.69 (23.07)	*F*(1, 200) = 254.86 (< 0.001)
	Stakeholders	75.53 (16.77)	22.13 (22.53)	*F*(1, 200) = 366.01 (< 0.001)
	Control: realism	3.96 (0.14)	5.08 (0.14)	*F*(1, 200) = 31.69 (< 0.001)
	Control: imaginable	4.73 (0.16)	5.18 (0.16)	*F*(1, 200) = 3.99 (0.047)
4	Long-term	73.77 (16.83)	32.34 (23.31)	*F*(1, 89) = 95.37 (< 0.001)
	Stakeholders	75.62 (20.24)	27.84 (24.34)	*F*(1, 89) = 104.19 (< 0.001)
	Control: realism	4.00 (1.81)	5.86 (1.13)	*F*(1, 89) = 34.23 (< 0.001)
5	Long-term	68.08 (20.19)	39.00 (21.81)	*F*(1, 139) = 64.47 (< 0.001)
	Stakeholders	64.74 (19.98)	40.44 (27.52)	*F*(1, 139) = 36.35 (< 0.001)
6	Long-term	53.95 (20.14)	43.82 (23 > 75)	*F*(1,88) = 4.78 (0.031)
	Stakeholders	50.11 (20.02)	36.27 (25.85)	*F*(1,88) = 5.93 (0.017)

A one-way between subjects ANOVA, controlling for realism, revealed that participants who saw the stakeholder-oriented firm humanized the stakeholders more (*M* = 5.10; *SD* = 1.04) than participants who saw the profit-oriented firm [*M* = 4.22; *SD* = 1.46; *F*(1,178) = 25.04; *p* < 0.001], thus supporting hypothesis 1.

Before the analysis, we removed participants based on two attention check questions. Rerunning the analysis including all participants revealed the same results: participants who saw the stakeholder-oriented firm humanized the stakeholders more than participants who saw the profit-oriented firm [*F*(1, 196) = 21.55; *p* < 0.001]. This supports that observing a stakeholder orientation causes humanization of stakeholders.

## Experiments 2–4

In experiment 1, we showed that participants who saw the stakeholder-oriented firm humanize other stakeholders in general more than participants who saw the profit-oriented firm. Experiments 2–4 aim to expand, specify, and generalize these findings. We expand our findings by also investigating participants’ moral consideration for other stakeholders. We aim to specify our findings by investigating if participants also humanize more specific stakeholders such as employees (exp. 2), suppliers (exp. 3) and labor unions (exp. 4). We aim to generalize our findings by investigating if the results generalize for different participant samples.

### Methods

#### Sample and Design

We expected large to moderate effect sizes (Rai and Diermeier, 2015), which requires a sample size of 59 to 124 for a percentile bootstrap test for mediation (Fritz and MacKinnon, 2007). In experiment 2, 205 participants recruited on a Dutch university campus completed the experiment online (97 men; *M*_*age*_ = 29.64; 104 stakeholder-oriented). After removing all participants who gave a wrong answer to an attention check, 183 participants remained (88 men; *M*_*age*_ = 30.95; 89 stakeholder-oriented). In experiment 3, 205 students completed the experiment for credit in a university lab (114 men, *M*_*age*_ = 25.62; 105 stakeholder-oriented). After removing all participants that gave a wrong answer to an attention check, 202 participants remained (113 men; *M*_*age*_ = 25.62; 102 stakeholder-oriented). In experiment 4, 97 participants (37 men; *M*_*age*_ = 25.78; 49 stakeholder-oriented) completed the experiment, of which 91 participants passed the attention checks (37 men; *M*_*age*_ = 25.84; 47 stakeholder-oriented). For experiment 4, we recruited participants via Prolific.ac and compensated them with 1 British Pound upon completion. Participants on Prolific have been found to be more honest and naïve about common psychological tasks than on Mturk (Peer et al., 2017). As in experiment 1, participants were randomly assigned to a stakeholder- or profit-oriented vignette, thus using a one-way between-subjects design. The vignette was followed by an attention check, and a survey probing for participants’ humanization of, and moral consideration for, respectively, employees, suppliers, and unions. The order of humanization and moral consideration was randomized. This was followed by a manipulation check, control variables, and demographics.

#### Procedure

The procedure started as in experiment 1. We adapted the vignettes of experiment 1 by adding a line about labor unions. The *stakeholder-oriented* (profit-oriented) line read:

Alpha also regularly *meets with* (refuses to meet with) labor unions, *even if these meetings are time-intensive and costly* (because these meetings are time-intensive and costly).

After the attention check, we measured humanization and moral consideration in randomized order. In order to measure moral consideration, we adapted and extended items from Agle et al. (1999) who measured perceived legitimacy of stakeholders’ interests. To do so, we first introduced participants to interests of the respective stakeholder, as described below:

Experiment 2: The interests of Alpha’s employees are, among other things, the following: fair number of holidays, fair wage, sick leave, good work-life balance, and motivated colleagues.

Experiment 3: The interests of Alpha’s suppliers are, among other things, the following: fair contracts, safe workplaces, economic development and innovation opportunities.

Experiment 4: The interests of Alpha’s labor unions are, among other things, the following: fair wages, safe working conditions, career development and educational opportunities for Alpha’s workers.

We then asked participants to indicate on a scale from 1 to 7 to what extent they agreed with the following statements: The interests of the employees (suppliers/labor unions): “are reasonable,” “deserve consideration,” “are in line with moral norms,” “deserve consideration regardless of their effects,” “should receive high priority,” and “should receive time and attention.” The scale’s reliability is high, with Cronbach’s alpha’s reaching 0.881 (exp. 2), 0.849 (exp. 3) and 0.933 (exp. 4).

### Results and Discussion

Table 1 summarizes the manipulation checks and controls of Experiments 2–4. In all three experiments, a MANOVA revealed that the stakeholder-oriented firm was perceived as significantly more long-term oriented than the profit-oriented firm and as significantly more prioritizing stakeholders than the profit-oriented firm. In experiment 2, participants exposed to the profit-oriented vignette were significantly older than participants exposed to the stakeholder-oriented vignette. In experiment 3, the profit-oriented vignette was perceived as significantly more realistic and imaginable than the stakeholder-oriented vignette, and in experiment 4, the profit-oriented vignette was perceived as significantly more realistic than the stakeholder-oriented vignette. We proceeded with the analysis but included age (exp. 2), both realism and imaginability (exp. 3), and realism (exp. 4), as a control variable.

**FIGURE 1:**
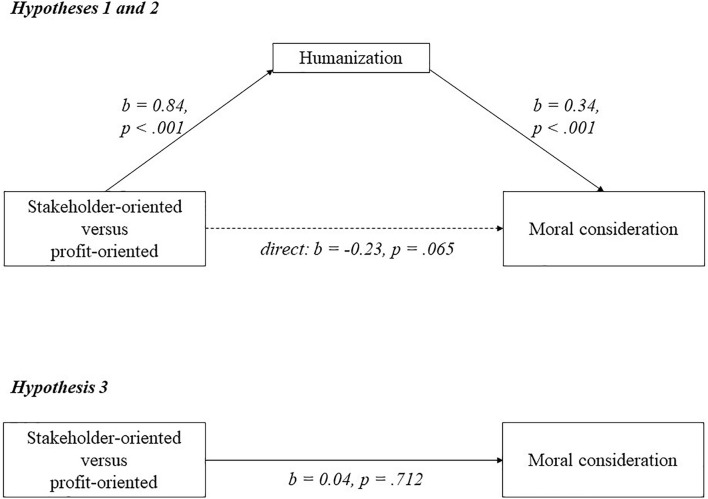
A model of mediation indicating that the relationship between business orientation and moral consideration goes via humanization in experiment 2.

**FIGURE 2:**
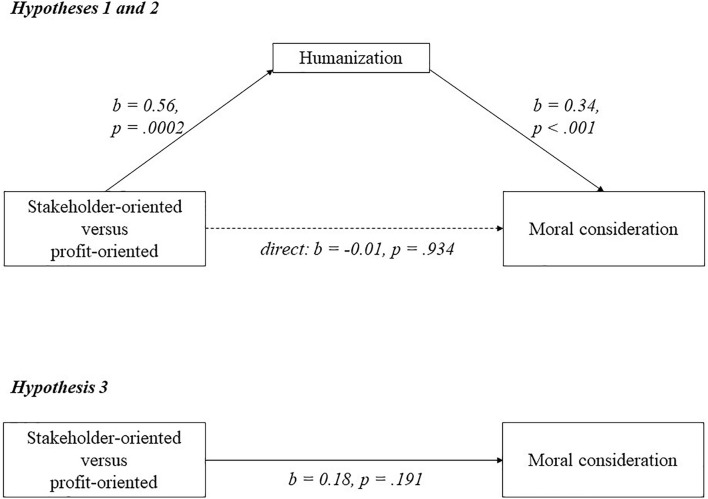
A model of mediation indicating that the relationship between business orientation and moral consideration goes via humanization in experiment 3.

**FIGURE 3:**
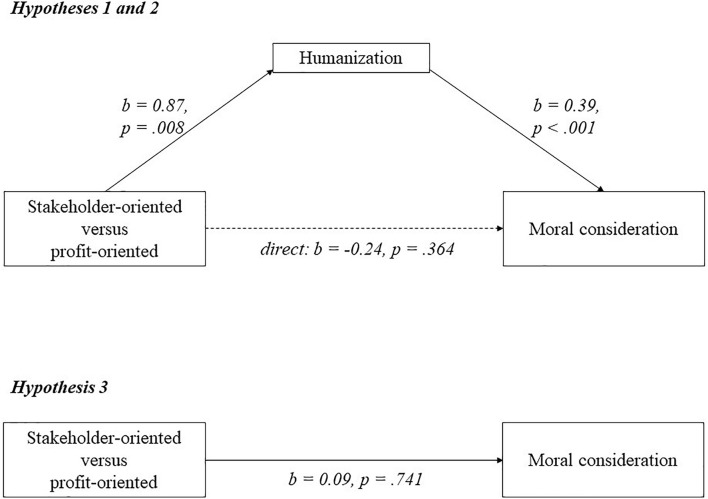
A model of mediation indicating that the relationship between business orientation and moral consideration goes via humanization in experiment 4.

Figures 1–3 visualize the results of experiments 2–4. For each experiment, a mediation analysis using model 4 of the PROCESS macro in SPSS (Hayes, 2013) revealed the same pattern. Participants who saw the stakeholder-oriented firm humanized the employees (*M* = 5.31; *SD* = 0.96), suppliers (*M* = 5.20; *SD* = 0.97) and labor unions (*M* = 5.53; *SD* = 0.97) compared to participants who saw the profit-oriented firm (employees: *M* = 4.48; *SD* = 1.08; suppliers: *M* = 4.68; *SD* = 1.00; labor unions: *M* = 4.68; *SD* = 1.58). As can be seen in Figures 1–3, these relations are significant (employees: *b* = 0.84; *p* < 0.001; suppliers: *b* = 0.56; *p* = 0.0002; labor unions: *b* = 0.87; *p* = 0.008), supporting hypothesis 1. Higher humanization of employees, suppliers, and labor unions was positively and significantly related to moral consideration for employees (*b* = 0.34; *p* < 0.001), suppliers (*b* = 0.34; *p* < 0.001) and labor unions (*b* = 0.39; *p* < 0.001), supporting hypothesis 2.

For all three experiments, participants did not show more moral consideration for employees (*M* = 5.69; *SD* = 0.83), suppliers (*M* = 5.38; *SD* = 0.82) or labor unions (*M* = 5.87; *SD* = 1.02) when they saw the stakeholder-oriented firm, than for employees (*M* = 5.63; *SD* = 0.84; *b* = 0.04; *p* = 0.712), suppliers (*M* = 5.21; *SD* = 0.96; *b* = 0.18; *p* = 0.191) or labor unions (*M* = 5.51; *SD* = 1.31; *b* = 0.09; *p* = 0.741) when they saw the profit-oriented firm. This seems to go against hypothesis 3, as illustrated in Figures 1–3. Further analysis shows that observing the more stakeholder-oriented firm indirectly increased moral consideration via humanization, and this indirect effect was significant for employees (*b* = 0.33; *LLCI* = 0.19; *ULCI* = 0.50), suppliers (*b* = 0.19; *LLCI* = 0.09; *ULCI* = 0.34) and labor unions (*b* = 0.34; *LLCI* = 0.13; *ULCI* = 0.69). Hypothesis 3 is therefore partially supported, because observing a stakeholder orientation increases moral consideration via humanization. Finally, the remaining direct effect was not significant for employees (*b* = -0.23; *p* = 0.065), suppliers (*b* = -0.01; *p* = 0.934) or labor unions (*b* = -0.24; *p* = 0.364). To check the robustness of the findings, we reran the analyses with all participants, and with and without the control variables. The results and significance levels reveal the same patterns.

While these results support hypotheses 1 and 2, they are also surprising because there is no total positive relationship between a stakeholder orientation and moral consideration. One possible explanation could be the randomized order of mediator and dependent variable. When asking participants about moral consideration *before* humanization (as was the case for about half the participants), participants might not explicitly take stakeholders’ human attributes into account when showing moral consideration. It is possible that moral consideration for stakeholders only increases when participants have explicitly humanized stakeholders; and this is more likely to happen when humanization is asked before moral consideration. In order to explore this possibility, we tested if the order of mediator and dependent variable moderated the relationships. These analyses showed that there was only one significant moderation effect: In the case of labor unions, when humanization was asked before moral consideration, the effect of business orientation on humanization was significantly more positive than when humanization was asked after moral consideration (*b* = 1.66; *p* = 0.002). This is in line with the possibility that humanization was not explicit enough when it was asked after moral consideration. To further investigate this possibility, we redid experiment 2, with the difference that humanization was now always asked before moral consideration.

## Experiment 5

### Methods

#### Sample, Design, and Procedure

One hundred sixty two participants from clickworker.com, who were compensated 0.7€, completed the experiment (91 men; *M*_*age*_ = 38.07; 81 stakeholder-oriented). After removing all participants who gave a wrong answer to an attention check, we retained 141 participants (78 men; *M*_*age*_ = 38.35; 73 stakeholder-oriented). The vignette was followed by an attention check, and a survey probing first for participants’ humanization of employees, and then for participants’ moral consideration for employees. In contrast to experiments 2-4, The order of the mediator and dependent variable was not randomized. This was followed by a manipulation check, control variables, and demographics.

### Results and Discussion

A MANOVA revealed that the stakeholder-oriented firm was perceived as significantly more long-term oriented (*M* = 68.08; *SD* = 20.19) than the profit-oriented firm [*M* = 39.00; *SD* = 21.81; *F*(1, 139) = 64.47; *p* < 0.001] and as significantly more prioritizing stakeholders (*M* = 64.74; *SD* = 19.98) than the profit-oriented firm [*M* = 40.44; *SD* = 27.51; *F*(1, 139) = 36.35; *p* < 0.001]. There were no differences in control variables or demographics between the vignettes.

**FIGURE 4:**
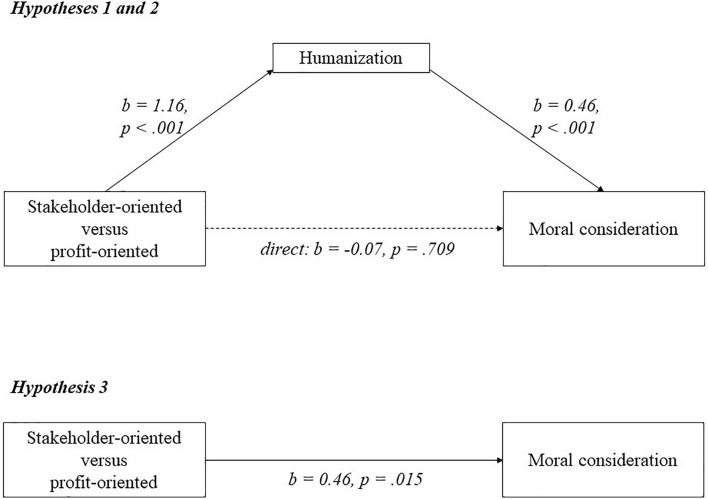
A model of mediation indicating that the relationship between business orientation and moral consideration goes via humanization in experiment 5.

As can be seen in Figure 4, Participants who saw the stakeholder-oriented firm humanized the employees (*M* = 5.39; *SD* = 0.87) compared to participants who saw the profit-oriented firm (*M* = 4.24; *SD* = 1.21; *b* = 1.16; *p* < 0.001), supporting hypothesis 1. Higher humanization of employees was positively and significantly related to moral consideration for employees (*b* = 0.46; *p* < 0.001), supporting hypothesis 2. Participants who observed the stakeholder-oriented firm showed more moral consideration for employees (*M* = 5.65; *SD* = 0.95) than participants who observed the profit-oriented firm (*M* = 5.19; *SD* = 1.29; *b* = 0.46; *p* = 0.015) and this effect was fully mediated by humanization (*b* = 0.54; *LLCI* = 0.274; *ULCI* = 0.842). This supports hypothesis 3. There was no remaining direct effect of stakeholder orientation on moral consideration (*b* = -0.07; *p* = 0.709).

While these results support hypotheses 1–3, one could argue that our stakeholder orientated firm may have increased participants’ moral consideration via other mechanisms. Stakeholder oriented firms may be perceived as promising a fair treatment of other stakeholders (Bosse et al., 2009), and this perceived fairness can make morality salient among individual stakeholders (Bridoux and Vishwanathan, 2020). This effect can also occur because a promise can be seen as morally binding (Haran, 2013). It is therefore important to note that the stakeholder-oriented vignette was explicitly described as “doing what is morally right” (versus being a successful business for the profit-oriented vignette). While explicitly mentioning the word “moral” did not result in a direct effect of business orientation on moral consideration in the previous experiments, it is possible that it influenced the indirect effect. To rule out this possibility, in the next experiment, we left out the word “moral.” As explained above, the vignettes now also focused on stakeholder (profit) elements without manipulating profit (stakeholder) elements.

## Experiment 6

In experiment 6, we aim to replicate our findings using different descriptions of the firm. We manipulate the firm’s orientation without a trade-off between stakeholder elements and profit elements. We also leave out explicit references to morality in the description.

### Methods

#### Sample and Design

One hundred and twelve participants from clickworker.com completed the experiment (57 men; *M*_*age*_ = 35.87; 57 stakeholder-oriented) and were compensated with 0.7€. After removing participants who gave a wrong answer to an attention check, 90 participants remained (45 men, *M*_*age*_ = 35.33, 46 stakeholder-oriented). Participants were randomly assigned to a stakeholder- or profit-oriented vignette, thus using a one-way between-subjects design.

#### Procedure

Participants were instructed to carefully read the description of a hypothetical grocery store Alpha. The description was the same as before, except that the *stakeholder-oriented* (profit-oriented) vignette only explicitly stated stakeholder (profit) elements. In addition, we left out the word “moral.” The *stakeholder-oriented* (profit-oriented) vignette now read:

Alpha is committed to improving its *stakeholders’ welfare* (financial performance) because Alpha believes this is *what is means to be a successful business* (necessary to be a successful business). This commitment to *stakeholder welfare and being a successful business* (financial performance and being a successful business) translates into practices that improve *stakeholder welfare* (financial performance). Specifically, Alpha *invests in relationships with its suppliers* (switches to the supplier who asks the lowest price). In addition, Alpha constantly optimizes its operations to increase *customer satisfaction* (profits). When new skills are needed, Alpha *trains its current employees* (replaces its employees with skilled applicants who ask the same wage). Finally, Alpha resolves conflicts with the local community *through collaboration* (*via* legal procedures).

The vignette was followed by an attention check, and a survey probing first for participants’ humanization of employees, and then for participants’ moral consideration for employees. As in experiment 5, the order of the mediator and dependent variable was not randomized. This was followed by a manipulation check, control variables, and demographics.

### Results and Discussion

A MANOVA revealed that the stakeholder-oriented firm was perceived as significantly more long-term oriented (*M* = 53.95; *SD* = 20.14) than the profit-oriented firm [*M* = 43.82; *SD* = 23.75; *F*(1,88) = 4.78; *p* = 0.031] and as significantly more prioritizing stakeholders (*M* = 50.11; *SD* = 20.02) than the profit-oriented firm [*M* = 38.27; *SD* = 25.85; *F*(1,88) = 5.93; *p* = 0.017]. There were no differences in the demographic or control variables between the vignettes.

**FIGURE 5:**
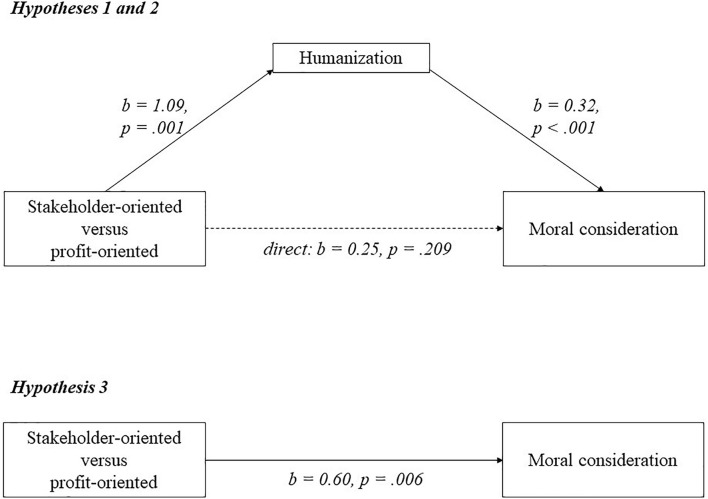
A model of mediation indicating that the relationship between business orientation and moral consideration goes via humanization in experiment 6.

As can be seen in Figure 5, a mediation analysis using model 4 of the PROCESS macro in SPSS (Hayes, 2013) revealed that participants who saw the stakeholder-oriented firm humanized the employees (*M* = 6.25; *SD* = 1.46) significantly more than participants who saw the profit-oriented firm (*M* = 5.15; *SD* = 1.61; *b* = 1.09; *p* = 0.001), supporting hypothesis 1. Higher humanization of employees, was positively and significantly related to moral consideration for employees (*b* = 0.32; *p* < 0.001). Participants showed more moral consideration for employees when they saw the stakeholder-oriented firm (*M* = 5.95; *SD* = 0.95) than when they saw the profit-oriented firm (*M* = 5.35; *SD* = 1.07; *b* = 0.60; *p* = 0.006), and this was mediated by humanization (*b* = 0.35; *LLCI* = 0.14; *ULCI* = 0.59). There was no remaining direct effect (*b* = 0.25; *p* = 0.209), supporting full mediation. To check the robustness of the findings, we reran the analyses with all participants, and without the control variables. The results and significance levels reveal the same patterns.

## Discussion and Conclusion

We investigated if business can humanize its stakeholders, and if so, how this humanization relates to moral consideration for stakeholders. We predicted and found that individual stakeholders observing a stakeholder-oriented firm humanize the firm’s other stakeholders compared to individual stakeholders observing a profit-oriented firm, and this humanization increases their moral consideration for the firm’s other stakeholders. We replicated this for different other stakeholders (employees, suppliers, and labor unions) and across different participant samples (students in a lab and online participants). We also predicted that individual stakeholders observing a stakeholder-oriented firm show more moral consideration for the firm’s other stakeholders than individual stakeholders observing a profit-oriented firm. This effect was only supported if individual stakeholders answered questions about humanization before answering questions about moral consideration. We replicated this for two operationalizations of a stakeholder and profit orientation. These findings strengthen the importance of a stakeholder orientation and humanization for increasing stakeholders’ moral consideration for each other.

This paper contributes to a deeper understanding of the firm as a moral community of stakeholders. Scholars from a variety of disciplines have argued that managers have a moral responsibility toward stakeholders (Freeman, 1984; Newkirk and Freeman, 2008; Freeman et al., 2010; Akrivou and Bradbury-Huang, 2015). However, stakeholders who do not have a managerial role also have a moral responsibility toward each other. This moral responsibility is important because stakeholders are interdependent and share a common goal with the firm and society (Phillips, 1997; Engster, 2011). In addition, stakeholders’ sense of moral responsibility is important to uphold or create moral and successful firms (Goodstein and Wicks, 2007; Elms and Phillips, 2009). For instance, consumers have a shared responsibility to reduce electronic waste that could harm other stakeholders in the value chain (Goodstein and Wicks, 2007). Another example is when a manufacturer retracts a damaging product, thereby benefitting customers, while at the same time disadvantaging shareholders (Liu et al., 2017). These shareholders should then show moral consideration for customers’ interests.

Previous work on stakeholder responsibility tends to be normative, and does not address how to increase stakeholders’ moral consideration for each other. We believe that integrating our empirical work can improve insights in how to cultivate responsible stakeholders. Specifically, we find a mechanism that increases individual stakeholders’ moral consideration in a way that transcends stakeholder groups: The same mechanism increases individual stakeholders’ moral consideration for employees, suppliers *and* labor unions. In addition, this replicated for participants on campus as well as online. This suggests that a stakeholder orientation, in situations that allow for explicit humanization, increases stakeholders’ moral consideration for each other.

This work supports and expands previous theorizing that proposes a link between a stakeholder orientation and moral consideration (Phillips et al., 2011; Bridoux and Vishwanathan, 2020). Scholars have focused on how teaching prevalent, profit-oriented, business theories to future managers causes morally disengaged management practices (Ferraro et al., 2005; Ghoshal, 2005). As an alternative, scholars suggest that teaching future managers more humanistic and ethical theories will lead to more morally engaged management practices (Newkirk and Freeman, 2008; Akrivou and Bradbury-Huang, 2015; Akrivou and Scalzo, 2020). While this is important, it begs the question if business can make individual stakeholders who are not managers more morally considerate.

The theories we teach future managers are no doubt important. However, our work shows that the business context itself, outside of a teaching context, also matters. Numerous studies show how business cues involving money reduce moral consideration (e.g., Beus and Whitman, 2017; Wang et al., 2020). When searching for an alternative to profit-oriented business, we decided to look at current developments where business leaders increasingly express their commitment to a stakeholder orientation. We find that a stakeholder orientation can increase stakeholders’ moral consideration for each other. This finding expands the links between a stakeholder orientation, humanization and moral consideration, from a teaching context to a business context.

Our work also unearths the causal role of humanization. We show that a stakeholder orientation only increases moral consideration if participants (cast in the role of individual stakeholder) are explicitly asked to attribute human qualities to the firm’s other stakeholders, before they are asked about their moral consideration. This shows that humanization is crucial for a stakeholder orientation to increase moral consideration. Some previous theorizing on the link between a stakeholder orientation and moral consideration has not considered the role of humanization (Phillips et al., 2011; Bridoux and Vishwanathan, 2020). Other previous theorizing has considered humanization, but does not explicate its causal role in fostering moral consideration (e.g., Purnell and Freeman, 2012). Our study suggests that a stakeholder orientation in itself does not increase moral consideration. Instead, a business context should also create situations where individual stakeholders explicitly reflect on other stakeholders’ human attributes.

### Limitations and Future Research

We recognize potential limitations of this paper. We made use of experiments in a lab or online with hypothetical descriptions of firms. A strength of controlled experiments is that they allow to investigate causality, isolate theoretical mechanisms, replicate findings and extend findings (Shadish et al., 2002; Haslam and McGarty, 2004; Aguinis and Bradley, 2014). A limitation of controlled experiments is that they sacrifice generalizability, compared to experiments in real settings (Aguinis and Bradley, 2014). Experiments therefore provide only an initial step to understand if there is a causal relationship, so that there can be more confidence when we subsequently test relationships in increasingly realistic settings.

Future research can investigate to what extent individuals see existing organizations as stakeholder-oriented or profit-oriented. It is an open question if organizations are intersubjectively perceived as stakeholder- or profit-oriented, or if there is instead much inter-individual variation. In addition, it is an open question which other factors increase perceptions that the firm is stakeholder-oriented. An analysis of perceptions of existing firms can shed light on this matter. In addition, it is an open question if individuals self-select on the basis of their own preferences and the firm’s orientation. Some individuals may be more attracted to stakeholder-oriented firms. If stakeholder-oriented firms attract stakeholder-oriented people, this can strengthen and stabilize the firm’s orientation.

Our work investigates individual stakeholders’ immediate reactions to an observed business orientation. While these immediate reactions are important, developing a moral community of stakeholders also requires a long-lasting transformation of individual stakeholders’ character or virtues (Akrivou and Scalzo, 2020). Laboratory experiments can disentangle the different psychological mechanisms that contribute to such a transformation. However, the development of virtuous character itself cannot be investigated in a laboratory experiment. We therefore suggest undertaking longitudinal studies, which assess individual stakeholders’ humanization, moral consideration, habits and virtues over time.

Another limitation of our study is that we focus only on human stakeholders as objects of humanization and moral consideration. Recently, there is a groundswell of support for the idea that animals are also stakeholders who deserve moral consideration (Engster, 2006; Merskin, 2021; Sayers et al., 2021; Smart, 2021; Tallberg et al., 2021). In addition, organizations put pressure on the Earth’s system, to the extent that the conditions for safe and just societies become undermined (Rockström et al., 2009; Whiteman et al., 2013); this development reinforces earlier arguments that nature is a stakeholder deserving moral consideration (Starik, 1995; Driscoll and Starik, 2004). In this light, an important question is how we can increase moral consideration for non-human stakeholders.

We do not expect our findings to extrapolate to all non-human stakeholders. First, it is an open question if non-human stakeholders can be humanized by describing the firm’s orientation toward these stakeholders. Typically, non-human stakeholders are humanized by other mechanisms, such as describing them as similar to humans (Bastian et al., 2012a). Another mechanism worthy of investigation might be to stress that firms, organizations and human societies are existentially dependent on certain non-human entities such as animals, a stable climate, or nature (Marcus et al., 2010). Second, it is an open question what moral consideration for non-human stakeholders would look like. It is possible that moral consideration for non-human stakeholders would fit a morality of care. Tronto (1993, p. 40) understands care as an activity we engage in “to maintain, continue, and repair our “world” so that we can live in it as well as possible.” Our measure of moral consideration focuses on stakeholders’ interests, but a care-based measure could focus on the needs or integrity of non-human stakeholders. We hope that future work also investigates non-human stakeholders.

In sum, our work has empirically demonstrated that a stakeholder orientation can increase individual stakeholders’ moral consideration, via humanization of other stakeholders. Future research can investigate how this plays out in existing firms, over time, and for non-human stakeholders.

## Data Availability Statement

The original contributions presented in the study are included in the article/Supplementary Material, further inquiries can be directed to the corresponding author/s.

## Ethics Statement

The studies involving human participants were reviewed and approved by SBE Research Ethics Review Board. The patients/participants provided their written informed consent to participate in this study.

## Author Contributions

KQ: conceptualization, methodology, validation, formal analysis, investigation, resources, data curation, writing – original draft, and project administration. JH: methodology and writing – review and editing. BP: conceptualization and writing – review and editing. IB: writing – review and editing. All authors contributed to the article and approved the submitted version.

## Conflict of Interest

The authors declare that the research was conducted in the absence of any commercial or financial relationships that could be construed as a potential conflict of interest.

## Publisher’s Note

All claims expressed in this article are solely those of the authors and do not necessarily represent those of their affiliated organizations, or those of the publisher, the editors and the reviewers. Any product that may be evaluated in this article, or claim that may be made by its manufacturer, is not guaranteed or endorsed by the publisher.
